# An unusual reason for an inguinal swelling: De Garengeot’s hernia

**DOI:** 10.1093/jscr/rjab083

**Published:** 2021-03-22

**Authors:** Barbara Sommerhalder, Reint Burger, Marco Bueter, Andreas Thalheimer

**Affiliations:** Department of General Surgery, Spital Männedorf, Männedorf, Switzerland; Department of General Surgery, Spital Männedorf, Männedorf, Switzerland; Department of General Surgery, Spital Männedorf, Männedorf, Switzerland; Department of Visceral and Transplantation Surgery, University Hospital of Zürich, Zürich, Switzerland; Department of General Surgery, Spital Männedorf, Männedorf, Switzerland; Department of Visceral and Transplantation Surgery, University Hospital of Zürich, Zürich, Switzerland

## Abstract

We present the case of a 71-year-old female with an inguinal swelling. Intra-abdominally the appendix was found in a femoral hernia sac (De Garengeot’s hernia). A laparoscopic transabdominal preperitoneal hernia repair procedure was performed with uneventful post-operative course.

Clinical presentation of this type of hernia is unspecific and often not to be distinguished from a common incarcerated hernia. Computed tomography can be helpful in obtaining a diagnosis, although the definite diagnosis is mostly found intraoperatively. As surgical options are numerous, there is no consensus on the most suitable one. A laparoscopic approach incorporates the benefit of a total abdominal overview and the possibility of standard procedures. If the appendix appears normal, the use of synthetic mesh is considered safe and an incidental appendectomy is not necessarily required.

## INTRODUCTION

De Garengeot’s hernia is defined as the presence of an appendix within a femoral hernia and was first described by a French surgeon called René Jacques Croissant de Garengeot in 1731 [1]. While the femoral hernia itself is rather uncommon and accounts for only 3–5% of all abdominal wall hernias, De Garengeot’s hernia occurs in ~1% of all femoral cases and therefore represents an extremely rare entity. In 0.08–0.13% of the events the appendix is acutely inflamed or perforated [2].

We herein report a case of a 71-year-old Caucasian female patient without appendicitis or incarceration and present a literature review. This work has been reported in line with the SCARE criteria.

## CASE REPORT

A 71-year-old woman presented to her general practitioner complaining about a non-painful swelling in the right groin area. Physical examination revealed a mass at the right inguinal area without any further abnormalities. No laboratory tests or imaging were obtained. An inguinal hernia was the tentative diagnosis. Due to the intake of oral anticoagulation, the surgery was delayed even further.

**Figure 1:**
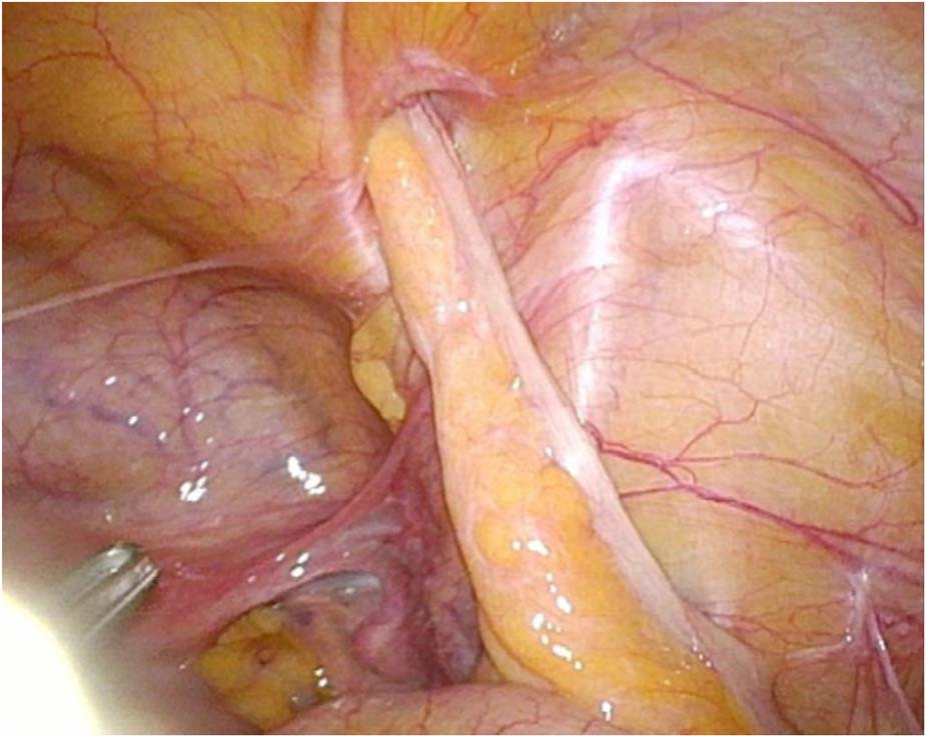
Laparoscopic view of a non-pathological appendix entering the femoral canal.

Under general anaesthesia, a transabdominal preperitoneal hernia repair (TAPP) was the procedure of choice. During the diagnostic laparoscopy, a femoral hernia with the appendix entering the femoral canal was detected (Fig. 1). The appendix showed no signs of inflammation or ischemia, but had adhesions to the peritoneum in the femoral sac. The hernia was repaired using a synthetic mesh; due to the lack of inflammation an appendectomy was not performed. The patient’s post-operative course was uneventful, and oral food intake was initiated on the first post-operative day. No unexpected events occurred, and the patient was discharged 48 h after the surgery.

## DISCUSSION

Femoral hernias constitute an uncommon type of groin hernias and account for only 3–5% of all abdominal wall hernias; they predominantly affect women over the age of 70 [3]. Femoral hernias have an increased risk of incarceration due to the narrow and rigid entrance to the femoral canal. Predisposing factors for incarceration may be a large appendix, a caecum positioned low in the pelvis or an abnormal intestinal rotation [2, 4, 5]. Our patient showed formation of adhesion of the appendix to the hernia sac. Priego *et al*. [6] suggested that if the appendix entered a femoral hernia sac by incidence it would be subject to successive local irritation and would eventually be retained there by adhesions. There is evidence supporting incarceration with subsequent ischemia and inflammation being the underlying cause for appendicitis and therefore possible gangrene and perforation [7].

Clinical presentation is variable and acute when incarceration is present. The most common clinical finding is a mass in the inguinal region, but signs of local inflammation (erythema, tenderness, warmth) can been found in a third of cases [3]. Due to the confined space of the narrow femoral canal, the inflammatory process is isolated and does rarely lead to peritonitis. Elevated laboratory tests have shown no association with longer duration of symptoms. Differential diagnosis should entail other forms of femoral hernia, inguinal hernia, lymphadenopathy, lymphadenitis, lipoma, abscess and venous ectasia/thrombophlebitis [8, 9].

As De Garengeot’s hernia is a rare entity, there is no consensus regarding the clinical and imaging management process. A pre-operative diagnosis can only be obtained in a third of the cases; diagnosis is most often made intraoperatively. Abdominal radiographs can be useful to exclude bowel obstruction but are of no further significance. A meta-analysis by Linder *et al*. [3] have shown that abdominal ultrasound might be helpful to rule out vascular component of the inguinal swelling but has only established the correct diagnosis in one case. With computed tomography (CT), a correct diagnosis can be achieved in almost 70% of cases; due to its rare nature, misinterpretation is a substantial risk and only experienced radiologists have shown to interpret the herniated content correctly as appendix [10].

However, in the acute setting the surgical exploration should never be delayed by extensive preoperative diagnostics.

As mentioned above, there is no recommendation regarding a specific surgical approach in the case of a De Garengeot’s hernia.

In general, most surgeons choose an open approach with access for both appendectomy and hernioplasty [3]. As an alternative serves the laparoscopic approach with the benefit of total abdominal overview and performance of standard laparoscopic procedures as appendectomy and/or TAPP [11]. If a pathological appendix is found the use of prosthetic material is generally not ideal as it can promote post-operative infection. Nevertheless, when a large hernia defect is discovered, the use of mesh is advised to avoid recurrence [12]. According to a number of studies the use of mesh in the absence of abscess formation or perforation did not result in infection [13].

According to Sharma *et al*. [14], incidental appendectomy is not required for a non-inflamed appendix. This conclusion is based on the concept that the removal of a contaminated organ in a clean contaminated wound violates aseptic principles of surgery. On the other hand, it may prevent repeat herniation, reduce the risk of future appendicitis and therefore need for another abdominal surgery [15].

In this case, the operation was an elective procedure with the assumptive diagnosis being an inguinal hernia, no visible signs of inflammation or ischemia intraoperatively. Repair by synthetic mesh was performed using a laparoscopic transabdominal preperitoneal (TAPP) approach.

In conclusion, the occurrence of De Garengeot’s hernia is indisputably low but has gained more attention in recent years as demonstrated by an increasing number of case reports. Therefore, it should be considered as differential diagnosis in patients presenting with acute onset of inguinal swelling with possible signs of local inflammation. Appropriate management without unnecessary delay caused by extensive imaging should lead to uneventful post-operative course. In case of large hernia defects or absence of abscess and perforation the use of a preperitoneal mesh is considered safe. An incidental appendectomy is not necessarily required when the appendix is not inflamed.

## AUTHOR CONTRIBUTION

Barbara Sommerhalder: data analysis and interpretation, writing and submission of the paper.

Reint Burger: interpretation of data.

Marco Bueter: interpretation of data, writing of the paper.

Andreas Thalheimer: interpretation of data, writing of the paper.

## CONFLICT OF INTEREST STATEMENT

None declared.

## FUNDING

The authors received no financial support for the research, authorship and/or publication of this article.

## ETHICAL APPROVAL

Exception from ethical approval-case report only, consent from the patient provided at request.

## CONSENT

Written informed consent was obtained from the patient for publication of this case report and accompanying images. A copy of the written consent is available for review by the Editor-in-Chief of this journal on request.

## GUARANTOR

Barbara Sommerhalder. Reint Burger. Marco Bueter. Andreas Thalheimer. Provenance and peer review. Not commissioned, externally peer-reviewed.
